# Weight-bearing cone-beam computed tomography in the foot and ankle specialty: where we are and where we are going - an update

**DOI:** 10.1590/0100-3984.2020.0048

**Published:** 2021

**Authors:** Alexandre Leme Godoy-Santos, Alessio Bernasconi, Marcelo Bordalo-Rodrigues, François Lintz, Carlos Felipe Teixeira Lôbo, Cesar de Cesar Netto

**Affiliations:** 1 Hospital das Clínicas da Faculdade de Medicina da Universidade de São Paulo (HC-FMUSP), São Paulo, SP, Brazil.; 2 Hospital Israelita Albert Einstein, São Paulo, SP, Brazil.; 3 Royal National Orthopaedic Hospital, Stanmore, United Kingdom.; 4 Clinique de l’Union, Saint Jean, Toulouse, France.; 5 Department of Orthopedics and Rehabilitation, Iowa City, Iowa, USA.

**Keywords:** Tomography, X-ray computed/methods, Cone-beam computed tomography, Weight-bearing, Foot/diagnostic imaging, Ankle/diagnostic imaging, Imaging, three-dimensional/methods, Tomografia computadorizada/métodos, Tomografia computadorizada de feixe cônico, Suporte de carga, Pé/diagnóstico por imagem, Tornozelo/diagnóstico por imagem, Imageamento tridimensional/métodos

## Abstract

Cone-beam computed tomography (CBCT) has been applied in dentistry and medicine for nearly two decades. Its application in the foot and ankle specialty has grown exponentially in recent years. Weight-bearing CBCT allows clinicians to obtain weight-bearing images that can be viewed in all three planes and to construct three-dimensional models, similar to those constructed from traditional CT scans, as well as exposing patients to less radiation than do traditional CT scans. This technology has revolutionized diagnoses, improving the understanding of various lesions and surgical planning in the foot and ankle specialty. Ongoing studies of the use of weight-bearing CBCT in foot and ankle surgery are focused on fully automated and semi-automated three-dimensional measurements, as well as bone segmentation, mapping of the distances/orientation of the joints, and the production of customized implants. The aims of this review article are to show the evolution of this emerging tool in the foot and ankle specialty, to update those in related specialties on its use in current clinical practice, and to indicate where the research community is heading.

## Abstract (pt)

A tomografia computadorizada (TC) de feixe cônico é aplicada na área da odontologia e medicina há cerca de duas décadas, e seu uso na especialidade do tornozelo e pé cresceu de forma exponencial nos últimos anos. A literatura demonstra como a TC de feixe cônico permite obter imagens com apoio do peso corporal total - *weight-bearing cone-beam computed tomography* - com cortes nos três planos e modelos tridimensionais semelhantes às TCs, associada a menor exposição à radiação, técnica que revolucionou o diagnóstico, o entendimento de diferentes lesões e o planejamento cirúrgico nessa área do conhecimento. As mensurações tridimensionais automáticas e semiautomáticas, a segmentação óssea, o mapeamento das distâncias e orientações articulares e a possibilidade de produção de implantes customizados são o interesse dos estudos em andamento na cirurgia do tornozelo e pé relacionados a essa ferramenta emergente. O objetivo deste artigo é mostrar a evolução do método, atualizar as especialidades de interface sobre uso na prática clínica atual e indicar para onde a comunidade científica está caminhando.

## INTRODUCTION

At the end of the 19th century, Wilhelm Conrad Roentgen claimed to have discovered something interesting, which allowed unprecedented developments in medicine in the decades that followed, through the use of conventional two-dimensional (2D) radiography, particularly an *in vivo* understanding of the osteoarticular apparatus. Technological advances have clearly shown that conventional radiography generated biased and somewhat distorted projections of the human body and did not provide a completely accurate representation of the anatomical reality^(1-4)^. Since the 1970s, computed tomography (CT) has furthered the *in vivo* study of the axial and appendicular skeleton, increasing the precision of three-dimensional (3D) assessments^(5)^ and providing greater diagnostic power to the vast majority of clinical and surgical specialties in the field of imaging research.

In the foot and ankle orthopedic specialty, conventional radiography and CT are essential tools for diagnosis, treatment planning, intraoperative control, and postoperative follow-up. Although conventional radiography allows one to study the foot and ankle in a weight-bearing standing position, its low spatial resolution and the superposition of anatomical structures generally prompt specialists to complement their diagnostic assessment with CT or magnetic resonance imaging (MRI). However, CT and MRI are performed with the patient in a horizontal position, with no load on the structures of the locomotor system, which significantly alters the relationships among the bones and joints in comparison with those seen in images acquired with the patient in the normal standing position.

For decades, physicians have been trained in the use of medical reasoning to integrate and apply conventional radiography, CT, and MRI in order to gather useful information about the anatomy, physiology, and pathology of their patients. Attempts to overcome the limits of each method have been detailed in the scientific literature. Several studies have documented the use of simulated weight-bearing CT with various types of devices. Unfortunately, such studies have only partially reproduced the condition of orthostatic support, because they have not involved muscle activation, which plays a fundamental role in determining the positions of the bones and their relationships with the joints^(6)^.

Cone-beam CT (CBCT) is utilized primarily in dental diagnostics and in the diagnostic assessment of the extremities. It involves the use of a cone-shaped X-ray beam and 2D detectors that acquire volume data with less rotation of the X-ray source. In conventional CT, the beams are fan-shaped and the detectors are one-dimensional. The advantages of CBCT include a shorter examination time, fewer motion artifacts, and the possibility of assessing the position of the foot and ankle. The disadvantages consist of an increase in scattered radiation and potential artifacts from the cone beam, which are similar to partial-volume artifacts.

Weight-bearing CBCT represents an important step forward in this technological evolution. It offers a consistent solution for acquiring images of the skeleton in the weight-bearing physiological standing position, which is crucial to understanding the deformities and degenerative lesions of the lower limbs^(1)^. In practice, weight-bearing CBCT combines the advantages of high spatial resolution 3D imaging with weight-bearing and muscle activation, as well as exposing patients to low doses of radiation (compared with CT), allowing the exact reproduction of dimensions and proportions. This innovation comes with challenges for the radiology community, because the existing tools applied to 2D radiography and conventional CT are not adapted to the new 3D environment. In addition, many measurements are still not fully automated, requiring greater involvement from radiologists and orthopedists in reading the exams. The use of weight-bearing CBCT offers individualized risk assessment and customized surgery, which are only beginning to become a reality.

## CONVENTIONAL RADIOGRAPHY AND ITS LIMITATIONS

In conventional radiography, the rotation component depends on the angle of incidence of the X-ray beam and changes in the rotation will result in different shapes and angles on the film. The distance between the X-ray source and the body can determine the projected lengths of bone structures, which can diverge from their actual lengths^(2,4)^.

Operator bias in conventional radiography is linked to technical aspects, the positioning of the X-ray equipment, and the positioning of the patient. In imaging of the foot and ankle, positioning of the foot in relation to the X-ray source, the height of the device, and the distances are difficult to reproduce with precision from one configuration to another. In practice, at least two X-rays are obtained for the same event in order to obtain an anteroposterior and a lateral view, which is the minimal acceptable combination in orthopedic imaging. However, images are never obtained twice in the same incidence, which would allow the assessment of reproducibility. In addition, the radiology technician may differ between examinations.

**Figure 1:**
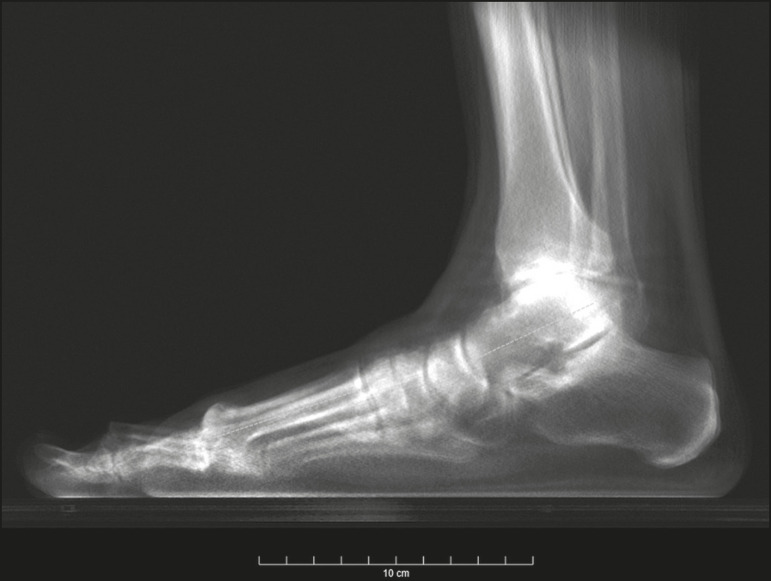
Lateral-view weight-bearing digital reconstructed radiograph of the ankle. Note the superposition and misalignment of the structures, leading to poor definition of the contours.

The superposition bias in conventional radiography, related to the projection of a 3D anatomical structure onto a 2D plane, where different structures are “stacked” in a single plane^(7)^, is more sensitive in the topography of the foot and ankle. This situation results in images with indistinguishable contours and overlapping edges (Figure 1), requiring considerable training for their correct interpretation.

Despite the aspects mentioned above, diagnostic imaging by conventional radiography with a load on the foot and ankle is widely applied in clinical practice and studied in the literature with a focus on intraobserver and interobserver reliability, thereby multiplying measurement techniques to improve the method^(8-11)^. The incidences of Saltzman view, Meary’s angle, long axial view, and hindfoot alignment angle exemplify the popularization of the use of this imaging option^(8-11)^. Although reproducible, the techniques are not accurate, because they do not correspond to the actual anatomical alignment, given that since they are based on the projection of the real structures of the foot and ankle. One recent study showed that when the foot is rotated 30° relative to the neutral position (focusing the X-ray tube in alignment with the second metatarsus), the measurement of the hindfoot alignment changes by 1% for each additional degree of rotation^(4)^. The average dispersion of radiographic measurements relative to the actual angle is approximately 20%^(12)^. Willauer et al. demonstrated how misalignment of the X-ray source of ≤ 25° in the transverse plane and ≤ 30° in the sagittal plane may lead to errors in the measurement of common angles, such as the calcaneal inclination and talonavicular coverage angles^(2)^. That has negative implications for the precision of surgical planning for corrective osteotomies in complex deformities. Therefore, it is evident that the acquisition of 3D weight-bearing images under the load of the total body weight is more reliable than is conventional bone imaging and provides a relevant benefit for the decision-making process with regard to treatment^(1,6,13)^.

## CBCT

### Technical characteristics

**Figure 2:**
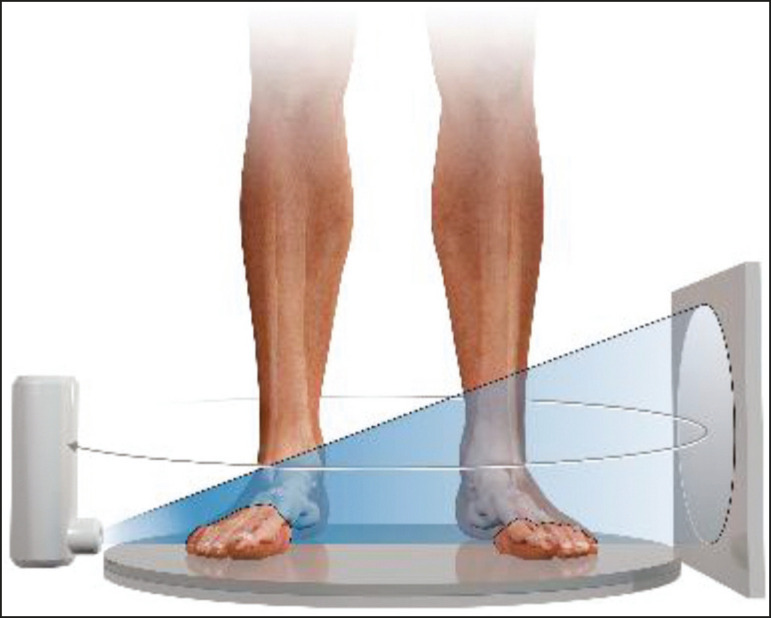
Illustration of image acquisition with weight-bearing CBCT. The patient stands in the center of the device. The source emits a cone-shaped X-ray beam (“cone beam”) captured by the flat-panel detector at the opposite end, while rotating on its axis.

On the basis of information from sequential 2D transverse slices captured by a standard flat-panel detector on the opposite end of the emission, CBCT allows the reconstruction of 3D models (Figure 2), involving the use of mathematical algorithms based on Radon and Fourier transforms^(14)^. The Fourier transform allows us to distinguish between several transverse slices, and the Radon transform allows us to calculate the coordinates of each pixel, so that we can reconstruct the entire volume slice by slice^(15)^.

### Exposure to radiation

In CBCT, the X-ray source performs only a single rotation around the anatomical target, whereas in conventional CT the X-ray source rotates around the target, in a spiral motion, multiple times. Therefore, CBCT exposes patients to a much lower radiation dose. The effective dose needed in order to acquire one view of the foot with conventional radiography is 0.001 mSv, compared with 0.07 mSv for one view of the ankle with conventional CT and 0.01-0.03 mSv for one view of the foot/ankle with CBCT^(6,13)^, the last corresponding to ≤ 1% of the mean annual radiation dose received by individuals in the United States (3 mSv) or to the equivalent of 10 conventional X-rays^(6,13)^. In weight-bearing CBCT, the total volume of a body segment is acquired in less than one minute. Richter et al.^(16)^ compared the radiation dose delivered during weight-bearing CBCT scans with those delivered during weight-bearing X-rays and conventional CT scans. The authors found that the mean annual dose was 0.5 µSv (10%) lower when weight-bearing CBCT was employed than when weight-bearing X-ray and conventional CT were employed (4.3 µSv vs. 4.8 µSv).

### Weight-bearing CBCT tools and learning curve

Understanding the weight-bearing CBCT model requires moving on from the former culture and secular learning required to understand 2D imaging to that of modern 3D imaging, which represents a roadblock to its adopting into the routine of the foot and ankle specialty. The reference points, positions, distances, and angles already defined for 2D imaging and consolidated in the literature are the initial bases for developing knowledge of 3D imaging, in which it is possible to define the position of each voxel in relation to an orthogonal frame. Currently, each voxel is defined by its X, Y, and Z coordinates and its density in Hounsfield units. The volume acquired in a typical bilateral weight-bearing CBCT contains 1,000,000 voxels, each approximately 0.3 × 0.3 × 0.3 mm in size. One foot is represented by approximately 200,000 voxels.

**Figure 3:**
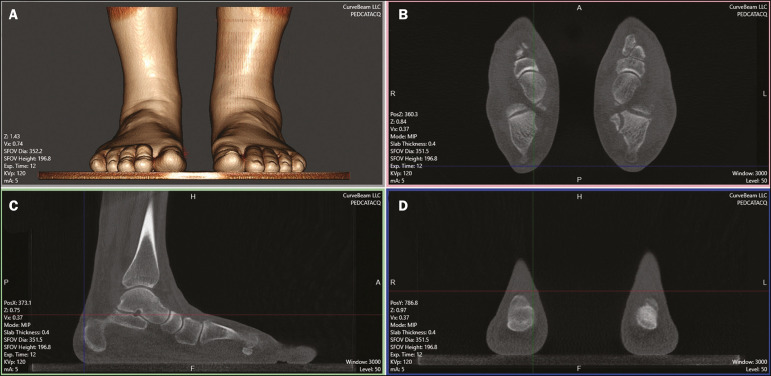
3D reconstruction (A) and multiplanar reconstructions (B,C,D), achieved with CubeVue software (CurveBeam LLC, Hatfield, PA, USA) after volumetric acquisition with weight-bearing CBCT.

**Figure 4:**
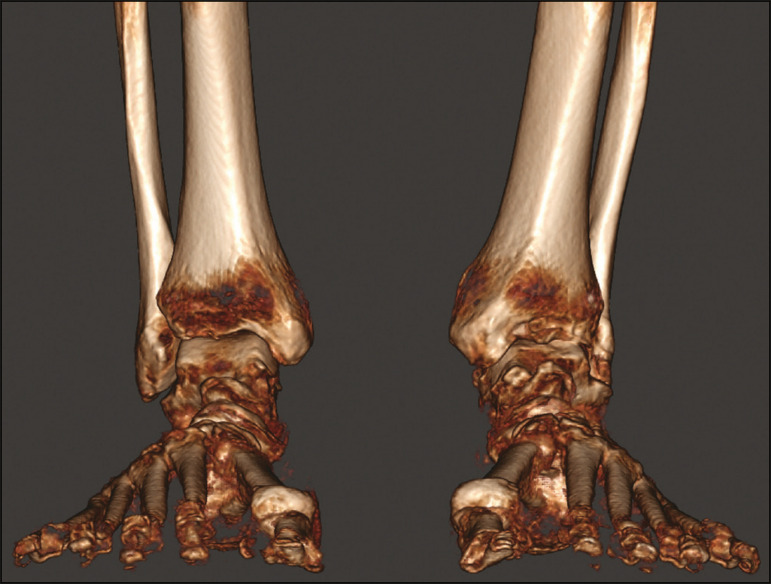
3D reconstruction for bone assessment, achieved with CubeVue software after volumetric acquisition with weight-bearing CBCT.

Most of the CT scanners currently available allow the creation of 3D multiplanar reconstructions (Figure 3). Digitally reconstructed radiographs (DRRs) are also obtained from the information contained in the 3D volume (Figure 4). These are radiographic reconstructions that are similar to traditional 2D images and can be created in any desired projection by applying computer-coordinated movements of the object of interest^(17,18)^. The advantage of DRRs over conventional X-rays is that DRRs make it possible to view multiple simulated radiographic projections from a single CT data set^(19)^. In research investigating foot and ankle pathologies, such as hallux valgus^(20)^, hindfoot alignment^(21)^, syndesmosis injuries^(22)^, and subtalar joint instability^(23)^, DRRs have been used in place of conventional X-rays in the calculation of angular measurements^(3)^. Multiplanar reconstructions and DRRs, both of which are obtained without increasing the radiation dose, serve as a gradual learning experience for a generation trained in the age of 2D imaging. They are also useful for obtaining an overview. Therefore, until an innovative solution is found, DRRs provide an interesting option (as opposed to older protocols that combine conventional CT and radiography).

### Cost-effectiveness

A typical weight-bearing CBCT scanner is still relatively expensive-approximately USD 150,000-250,000-weighs approximately 250 kg, and fits into an area of 1 × 1 m. Although there is still no precise cost analysis, the shorter acquisition time of weight-bearing CBCT-less than a minute-has resulted in a significant decrease in the number of appointments for conventional CT in radiology sectors and greater productivity for each scanner and technician involved^(1)^.

Martinus Richter was a pioneer in the use of 3D imaging in orthopedics, having described radioscopy, two-plane intraoperative measurement of plantar pressure, and weight-bearing CBCT^(24-27)^. In one recent, Richter et al.^(16)^ evaluated a collective sample of over 11,000 weight-bearing CBCT examinations performed over 5 years, reporting that imaging times were 77% shorter for weight-bearing CBCT examinations than for weight-bearing X-ray and conventional CT examinations.

## INDICATIONS FOR WEIGHT-BEARING CBCT

The literature on weight-bearing CBCT is mainly related to the investigation of pes planus, or flatfoot^(28)^; ankle fractures and syndesmosis injuries^(29,30)^; subtalar joint instability^(31,32)^; hypermobility of the first ray with hallux valgus^(33)^; hallux rigidus^(34)^; and patellar instability and tibiofemoral arthritis^(35,36)^.

Regarding flatfoot and subtalar joint instability, measurements analogous to those obtained with conventional radiology can be obtained with weight-bearing CBCT, which is better at determining severity^(28)^. Patients with flatfoot have more innate valgus in their talar shape and subtalar alignment^(31)^. In such patients, the fifth metatarsal bone demonstrates greater plantar flexion in relation to the first metatarsal bone in comparison with individuals without flatfoot^(32)^. Abnormal orientation of the subtalar joint is a potential risk factor for the development of osteoarthritis in the ankle joint^(31,32)^. Patients with hallux rigidus present with metatarsus primus elevatus (elevation of the first ray), which increases as the condition worsens^(34)^, together with increased mobility of the first tarsometatarsal joint, as well as of all other joints of the first ray^(33)^.

In individuals with tibiofibular syndesmosis, there is internal rotation of the talus in the varus osteoarthritic ankle, the rotation increasing as the condition worsens. With a load equivalent to the total body weight, the rotation of the talus in relation to the ankle is nearly 10°, the posterior translation of the fibula is 1.5 mm, and the external rotation is 3°. Its comparison with the contralateral side seems to be more reliable than its comparison with the population without injury^(37,38)^. In a recent study of patients with uninjured, asymptomatic ankles^(39)^, there were no significant differences between weight-bearing CBCT and conventional CT in terms of the measurements of the distal tibiofibular joint. However, the medial free space of the tibiotalar joint was apparently larger on the weight-bearing CBCT scans than on the conventional CT scans, which is probably due to the anterior displacement of the talus in the non-weight-bearing position, which is greater in the anterior portion than in the posterior portion. In patients with hallux valgus, determining the role of coronal rotation of the first metatarsal and sesamoid bones is essential to planning the correction of the deformity^(40-43)^.

### Role in preoperative planning

The use of weight-bearing CBCT allows a personalized assessment devoid of the variables inherent to conventional imaging^(6,44)^, thus facilitating the decision-making process regarding treatment. The subjectivity of clinical evaluations required that distances and angles based on 2D imaging be developed and used for preoperative orthopedic planning. These measurements represented a more reliable way of standardizing deformities by level of severity and made it possible to stratify patients, as well as to quantify postoperative outcomes. Some authors have shown that traditional 2D measurements can be used with greater precision in 3D imaging, especially in conjunction with new parameters and measurements^(28-38)^. To improve accuracy, there are three recommended ways to measure angles and distances in weight-bearing CBCT data sets: in the DRR mode^(44,45)^; in a single plane^(28-30)^; and in the total volume in 3D^(46)^.

**Figure 5:**
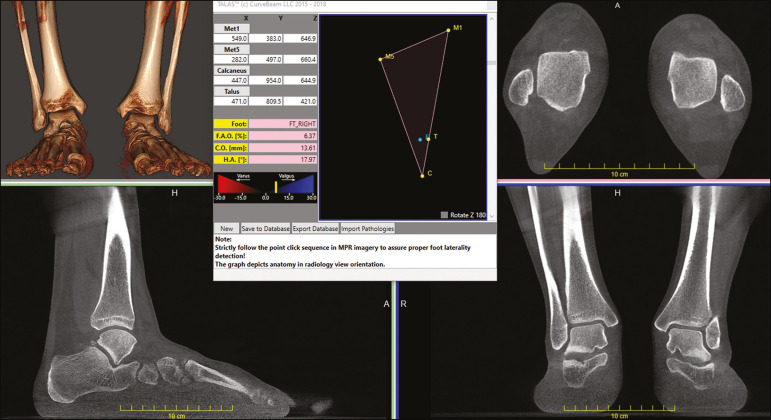
User interface demonstrating the application of the TALAS system (CurveBeam LLC) to calculate the foot ankle offset.

Traditional measurements of hindfoot alignment generally define the position of calcaneal projection through its position relative to the anatomical axis of the tibia. Saltzman et al.^(8)^ proposed replacing the tibial-calcaneal angle with a displacement, whereas Arunakul et al.^(47)^ and Lintz et al.^(12)^ proposed defining the position of the hindfoot in relation to the forefoot instead of the tibia. Subsequently, Lintz et al.^(48)^ developed a 3D biometric tool to measure hindfoot alignment-the foot ankle offset-which is applied with specific software (TALAS; CurveBeam LLC) and incorporated into the weight-bearing CBCT system (Figure 5). The tool is defined by three main characteristics-computerized, semi-automated, and volumetric-and is based on at least four reference points. The foot ankle offset tool determines the central position of the ankle joint in relation to the three main real weight-bearing points of the foot (the plantar apexes of the first and fifth metatarsal heads and the inferior calcaneal tuberosity). Better than an angle, a displacement has the advantage of being directly related to the lever arm and to the torque generated by the distance between the body weight applied to the ankle and the ground reaction force applied to the foot^(12,48)^. Some authors have shown that weight-bearing CBCT produces good quality images and has high reliability for common measurements^(1,28)^, as well as providing better soft tissue differentiation than that achieved with conventional CT^(49)^.

### Advantages and limitations of weight-bearing CBCT

The involvement of a new technology such as weight-bearing CBCT challenges us to look for fundamentals that support its advantages and disadvantages. In addition, given its high cost, it is essential to perform a comparative analysis with the available alternatives such as CT with a simulated load, conventional CT in the supine position, and conventional radiography. The main advantages of weight-bearing CBCT include the following: the possibility of using the classic radiographic angles and distances^(28,30,34)^; accurate rotation and axis recognition by software; radiation dose lower than that of conventional CT and similar to that of a complete X-ray series of the foot and ankle, including anteroposterior, lateral, oblique, and hindfoot alignment incidences^(6,44)^; image resolution comparable to that of conventional CT in a weight-bearing standing position under a total body weight load and with muscle activation, which replicates the actual condition of the anatomical structures under the force of gravity^(1,6)^; the total volume of the target is acquired in less than one minute; only a small physical area is needed in order to install the equipment; and software designed to suppress biases related to 2D measurements is available^(48)^. The use of weight-bearing CBCT also has a number of limitations: it is not yet widely available in Brazil, having been on the market only since 2014; the initial investment is high, and there has yet to be a cost-effectiveness analysis of the methodology; the measurements are still not fully automated, despite the fact that the method has shown excellent reliability^(48)^; and the software is not yet capable of accurately recognizing bone edges, because of the presence of osteophytes or severe degenerative changes^(46)^.

Given the quantity and quality of the evidence related to the use of weight-bearing CBCT in the foot and ankle specialty^(6,13,42)^, we believe that it is viable alternative to conventional radiography in imaging research protocols for the lower limbs. It is evident that clinical research plays a central role in demonstrating the potential of this method in clinical and surgical practice, thus reducing biases in interpretation and increasing accuracy in the recognition of deformities^(2-4,12)^.

Axial passive loading devices have been used in research based on simulated weight-bearing conditions in CT exams. Kang et al.^(50)^ designed a device to apply a passive axial load in 3D CT images acquired in the supine position and tested the device in a sample of 80 patients. They performed common measurements applied in the evaluation of hallux valgus and flatfoot, comparing the results with those obtained in weight-bearing X-rays. When the authors applied an axial load ≥ 70% of the body weight, they found no statistically significant difference between the two imaging modalities. However, the limitations of a simulated load should be considered. The use of devices that do not apply an accurate full weight-bearing load or apply only a partial load may result in an underestimation of the pathologies that would be observed in the standing position, and analyses of the results thus obtained do not take into consideration the impact that active muscle contraction has on foot and ankle alignment^(50)^. The simulation devices are custom-made and are not standardized, which may not reproduce the natural biomechanical standing position.

Future perspectives for weight-bearing CBCT include the development of measurement systems that are fully automated and reliable, which would eliminate the need for manual interventions, reduce the reading time per examination, and improve reproducibility^(48)^. In addition, the development of automated segmentation of the foot and ankle bones through complex algorithms will make it possible to calculate axes and relative orientations automatically. Another field in development is distance mapping^(46)^, which will allow one to study the interaction of the articular surface in a non-invasive manner by using 3D bone models collected from 3D images. In a recent study, Siegler et al.^(46)^ demonstrated how distance mapping provides detailed information about the ankle and subtalar joints during normal joint movements. Finally, the 3D models of weight-bearing CBCT images can be used in order to manufacture customized instruments and implants, thereby increasing the precision of the final products and likely improving postoperative functional outcomes.

The advent of weight-bearing CBCT brings new challenges. The angular measurements should be standardized on the basis of the identification on reliable anatomical landmarks and compared with those obtained with conventional weight-bearing X-rays, in order to establish the normal values, as well as the intraobserver and interobserver reliability^(6)^. Further prospective comparative studies are needed in order to translate the information acquired in this new 3D environment into information that is relevant and useful in the clinical setting.

## CONCLUSION

The weight-bearing CBCT technique allows us to obtain reliable 3D weight-bearing images of the foot and ankle while they are supporting the total body weight against the force of gravity and with muscle activation. The technique exposes patients to relatively low doses of radiation and provides excellent image resolution, as well as showing satisfactory interobserver and intraobserver reliability. Further studies are needed in order to determine its cost-benefit ratio and its impact on clinical and surgical outcomes. Current research efforts are aimed at evaluating new 3D measurements, developing fully automated bone segmentation systems, applying distance mapping techniques, and validating the use of 3D weight-bearing CBCT reconstructions as models for custom-made implants.

## References

[1] Richter M, Seidl B, Zech S (2014). PedCAT for 3D-imaging in standing position allows for more accurate bone position (angle) measurement than radiographs or CT. Foot Ankle Surg.

[2] Willauer P, Sangeorzan BJ, Whittaker EC (2014). The sensitivity of standard radiographic foot measures to misalignment. Foot Ankle Int.

[3] Barg A, Amendola RL, Henninger HB (2015). Influence of ankle position and radiographic projection angle on measurement of supramalleolar alignment on the anteroposterior and hindfoot alignment views. Foot Ankle Int.

[4] Baverel L, Brilhault J, Odri G (2017). Influence of lower limb rotation on hindfoot alignment using a conventional two-dimensional radiographic technique. Foot Ankle Surg.

[5] Ambrose J, Hounsfield G (1973). Computerized transverse axial tomography. Br J Radiol.

[6] Barg A, Bailey T, Richter M (2018). Weightbearing computed tomography of the foot and ankle: emerging technology topical review. Foot Ankle Int.

[7] Perlepe V, Omoumi P, Larbi A (2018). Can we assess healing of surgically treated long bone fractures on radiograph?. Diagn Interv Imaging.

[8] Saltzman CL, el-Khoury GY (1995). The hindfoot alignment view. Foot Ankle Int.

[9] Reilingh ML, Beimers L, Tuijthof GJM (2010). Measuring hindfoot alignment radiographically: the long axial view is more reliable than the hindfoot alignment view. Skeletal Radiol.

[10] Williamson ER, Chan JY, Burket JC (2015). New radiographic parameter assessing hindfoot alignment in stage II adult-acquired flatfoot deformity. Foot Ankle Int.

[11] Dagneaux L, Moroney P, Maestro M (2019). Reliability of hindfoot alignment measurements from standard radiographs using the methods of Meary and Saltzman. Foot Ankle Surg.

[12] Lintz F, Barton T, Millet M (2012). Ground reaction force calcaneal offset: a new measurement of hindfoot alignment. Foot Ankle Surg.

[13] Godoy-Santos AL, de Cesar Netto C, Weight-bearing CT, International Study Group (2018). Weight-bearing computed tomography of the foot and ankle: an update and future directions. Acta Ortop Bras.

[14] Mozzo P, Procacci C, Tacconi A (1998). A new volumetric CT machine for dental imaging based on the cone-beam technique: preliminary results. Eur Radiol.

[15] Scarfe WC, Farman AG (2008). What is cone-beam CT and how does it work?. Dent Clin North Am.

[16] Richter M, Lintz F, de Cesar Netto C (2020). Results of more than 11,000 scans with weightbearing CT - impact on costs, radiation exposure, and procedure time. Foot Ankle Surg.

[17] Bethune C, Stewart AJ (2005). Accelerated computation of digitally reconstructed radiographs. International Congress Series.

[18] Nelson V, Deshpande S, Gray A (2014). Comparison of digitally reconstructed radiographs generated from axial and helical CT scanning modes: a phantom study. Australas Phys Eng Sci Med.

[19] Lenz AL, Krähenbühl N, Howell K (2019). Influence of the ankle position and X-ray beam angulation on the projection of the posterior facet of the subtalar joint. Skeletal Radiol.

[20] de Cesar Netto C, Richter M (2020). Use of advanced weightbearing imaging in evaluation of hallux valgus. Foot Ankle Clin.

[21] Burssens ABM, Buedts K, Barg A (2020). Is lower-limb alignment associated with hindfoot deformity in the coronal plane? A weightbearing CT analysis. Clin Orthop Relat Res.

[22] Krähenbühl N, Bailey TL, Weinberg MW (2019). Impact of torque on assessment of syndesmotic injuries using weightbearing computed tomography scans. Foot Ankle Int.

[23] Krähenbühl N, Burssens A, Davidson NP (2019). Can weightbearing computed tomography scans be used to diagnose subtalar joint instability? A cadaver study. J Orthop Res.

[24] Richter M, Frink M, Zech S (2006). Intraoperative pedography: a validated method for static intraoperative biomechanical assessment. Foot Ankle Int.

[25] Richter M, Zech S, Leonard J (2009). Goldner award 2009 intraoperative pedobarography leads to improved outcome scores: a level I study. Foot Ankle Int.

[26] Richter M, Zech S, Hahn S (2016). Combination of pedCAT® for 3D imaging in standing position with pedography shows no statistical correlation of bone position with force/pressure distribution. J Foot Ankle Surg.

[27] Richter M, Lintz F, Zech S (2018). Combination of pedCAT weightbearing CT with pedography assessment of the relationship between anatomy-based foot center and force/pressure-based center of gravity. Foot Ankle Int.

[28] de Cesar Netto C, Schon LC, Thawait GK (2017). Flexible adult acquired flatfoot deformity: comparison between weight-bearing and non-weight-bearing measurements using cone-bean computed tomography. J Bone Joint Surg Am.

[29] Lawlor MC, Kluczynski MA, Marzo JM (2018). Weight-bearing cone-beam CT scan assessment of stability of supination external rotation ankle fractures in a cadaver model. Foot Ankle Int.

[30] Osgood GM, Shakoor D, Orapin J (2019). Reliability of distal tibio-fibular syndesmotic instability measurements using weightbearing and non-weightbearing cone-beam CT. Foot Ankle Surg.

[31] Probasco W, Haleem AM, Yu J (2015). Assessment of coronal plane subtalar joint alignment in peritalar subluxation via weight-bearing multiplanar imaging. Foot Ankle Int.

[32] Krähenbühl N, Tschuck M, Bolliger L (2016). Orientation of the subtalar joint: measurement and reliability using weightbearing CT scans. Foot Ankle Int.

[33] Kimura T, Kubota M, Taguchi T (2017). Evaluation of first-ray mobility in patients with hallux valgus using weight-bearing CT and a 3-D analysis system: a comparison with normal feet. J Bone Joint Surg Am.

[34] Cheung ZB, Myerson MS, Tracey J (2018). Weight bearing CT scan assessment of foot alignment in patients with hallux rigidus. Foot Ankle Int.

[35] Thawait GK, Demehri S, AlMuhit A (2015). Extremity cone-beam CT for evaluation of medial tibiofemoral osteoarthritis: initial experience in imaging of the weight-bearing and non-weight-bearing knee. Eur J Radiol.

[36] Marzo J, Kluczynski M, Notino A (2016). Comparison of a novel weightbearing cone beam computed tomography scanner versus a conventional computed tomography scanner for measuring patellar instability. Orthop J Sports Med.

[37] Lepojärvi S, Niinimäki J, Pakarinen H (2016). Rotational dynamics of the talus in a normal tibiotalar joint as shown by weight-bearing computed tomography. J Bone Joint Surg Am.

[38] Lepojärvi S, Niinimäki J, Pakarinen H (2016). Rotational dynamics of the normal distal tibiofibular joint with weight-bearing computed tomography. Foot Ankle Int.

[39] Shakoor D, Osgood GM, Brehler M (2019). Cone-beam CT measurements of distal tibio-fibular syndesmosis in asymptomatic uninjured ankles: does weight-bearing matter?. Skeletal Radiol.

[40] Lamo-Espinosa JM, Flórez B, Villas C (2015). The relationship between the sesamoid complex and the first metatarsal after hallux valgus surgery without lateral soft-tissue release: a prospective study. J Foot Ankle Surg.

[41] Geng X, Zhang C, Ma X (2016). Lateral sesamoid position relative to the second metatarsal in feet with and without hallux valgus: a prospective study. J Foot Ankle Surg.

[42] Chen JY, Rikhraj K, Gatot C (2016). Tibial sesamoid position influence on functional outcome and satisfaction after hallux valgus surgery. Foot Ankle Int.

[43] Welck MJ, Singh D, Cullen N (2018). Evaluation of the 1st metatarso-sesamoid joint using standing CT - the Stanmore classification. Foot Ankle Surg.

[44] Lintz F, de Cesar Netto C, Barg A (2018). Weight-bearing cone beam CT scans in the foot and ankle. EFORT Open Rev.

[45] Moore CS, Wood TJ, Saunderson JR (2017). A method to incorporate the effect of beam quality on image noise in a digitally reconstructed radiograph (DRR) based computer simulation for optimisation of digital radiography. Phys Med Biol.

[46] Siegler S, Konow T, Belvedere C (2018). Analysis of surface-to-surface distance mapping during three-dimensional motion at the ankle and subtalar joints. J Biomech.

[47] Arunakul M, Amendola A, Gao Y (2013). Tripod index: a new radiographic parameter assessing foot alignment. Foot Ankle Int.

[48] Lintz F, Welck M, Bernasconi A (2017). 3D biometrics for hindfoot alignment using weightbearing CT. Foot Ankle Int.

[49] Lechuga L, Weidlich GA (2016). Cone beam CT vs. fan beam CT: a comparison of image quality and dose delivered between two differing CT imaging modalities. Cureus.

[50] Kang DH, Kang C, Hwang DS (2019). The value of axial loading three dimensional (3D) CT as a substitute for full weightbearing (standing) 3D CT: comparison of reproducibility according to degree of load. Foot Ankle Surg.

